# Developing National Standards and Best Practices for Nursing Home Registered Apprenticeship Programs

**DOI:** 10.1093/geroni/igaf122.1984

**Published:** 2025-12-31

**Authors:** Robyn Stone, Molly Carpenter

**Affiliations:** Leading Age, Washington, District of Columbia, United States; The LeadingAge LTSS Center at University of Massachusetts, Washington, District of Columbia, United States

## Abstract

Under the Biden Administration, registered apprenticeships emerged as a promising strategy for healthcare providers to recruit, train, and retain a qualified workforce, supported by expanded access to federal and state funding. Yet, nursing homes and other aging services providers have been slow to adopt this model, often facing barriers related to regulatory complexity, limited infrastructure and resources, and lack of tailored guidance. This paper describes how the federally funded Geriatrics Workforce Enhancement Program (GWEP) is working to close this gap by supporting the development of registered apprenticeship programs for direct care professionals in long-term care settings. First, we outline the structure and distinguishing features of registered apprenticeships and describe how GWEP grantees are helping introduce and adapt this model within nursing homes. Second, we present the formation and early activities of a national GWEP learning network convened by the Moving Forward Nursing Home Quality Coalition and focused on developing common standards and best practices for apprenticeship implementation for nursing home certified nursing assistants (CNAs). Drawing on stakeholder input and early field experiences, we highlight considerations related to program design, partnership building, and alignment with quality improvement and other organizational goals. Finally, we discuss the broader policy and regulatory implications of expanding apprenticeships in the long-term care workforce and offer recommendations to support their sustainable growth.

